# Sleep education during pregnancy for new mothers

**DOI:** 10.1186/1471-2393-12-155

**Published:** 2012-12-17

**Authors:** Liora Kempler, Louise Sharpe, Delwyn Bartlett

**Affiliations:** 1 Woolcock Institute of Medical Research, The University of Sydney, 431 Glebe Point Rd, Glebe, Australia; 2 School of Psychology, The University of Sydney, Brennan MacCallum Building (A18), Sydney, Australia; 3 Brain and Mind Research Institute, The University of Sydney, 100 Mallett Street, Camperdown, Australia

**Keywords:** Sleep, New mothers, Postnatal, Postpartum, Depression, Psychoeducation

## Abstract

**Background:**

There is a high association between disturbed (poor quality) sleep and depression, which has lead to a consensus that there is a bidirectional relationship between sleep and mood. One time in a woman’s life when sleep is commonly disturbed is during pregnancy and following childbirth. It has been suggested that sleep disturbance is another factor that may contribute to the propensity for women to become depressed in the postpartum period compared to other periods in their life. Post Natal Depression (PND) is common (15.5%) and associated with sleep disturbance, however, no studies have attempted to provide a sleep-focused intervention to pregnant women and assess whether this can improve sleep, and consequently maternal mood post-partum. The primary aim of this research is to determine the efficacy of a brief psychoeducational sleep intervention compared with a control group to improve sleep management, with a view to reduce depressive symptoms in first time mothers.

**Method:**

This randomised controlled trial will recruit 214 first time mothers during the last trimester of their pregnancy. Participants will be randomised to receive either a set of booklets (control group) or a 3hour psychoeducational intervention that focuses on sleep. The primary outcomes of this study are sleep-related, that is sleep quality and sleepiness for ten months following the birth of the baby. The secondary outcome is depressive symptoms. It is hypothesised that participants in the intervention group will have better sleep quality and sleepiness in the postpartum period than women in the control condition. Further, we predict that women who receive the sleep intervention will have lower depression scores postpartum compared with the control group.

**Discussion:**

This study aims to provide an intervention that will improve maternal sleep in the postpartum period. If sleep can be effectively improved through a brief psychoeducational program, then it may have a protective role in reducing maternal postpartum depressive symptoms.

**Registration details:**

This trial is registered with the Australian New Zealand Clinical Trials Register under the registration number ACTRN12611000859987

## Background

On average, one third of a person’s life is spent asleep [1]. Sleep is vital, allowing the body to rest, replenish resources in the brain and body [2], and consolidate new information [3]. Since the 1960s, sleep duration has declined [4,5], complaints about sleep problems have almost doubled [6] and many individuals report poor sleep quality or consistently insufficient sleep at night [7]. For example, approximately 30% of the Australian population report sleeping for less than 6.5 hours per night [8]. Partial sleep deprivation (consistent short sleep) in humans is associated with hypertension, impairment of glucose control, weight gain [7], reduced concentration, irritability and depression [9]. The various consequences become apparent at different stages of partial sleep deprivation, with reduced cognitive performance and poor mood appearing after just one night of less than 5 hours of sleep [9]. Further, untreated sleep disturbance is known to lead to depression while depression can also precipitate sleep disturbance [10]. It is currently unclear whether disturbances to sleep most often precede mood disturbance or whether depression typically precedes sleep disturbance. There is consistent evidence to indicate that irrespective of the order of onset, sleep disturbance and depression are highly associated [11,12]. Sleep disruption is very common in depression and is one of the symptoms described by the Diagnostic and Statistical Manual of Mental Disorders – fourth edition in the diagnosis of depression [13]. Depression has also been shown to precede sleep disturbance and that with increased insomnia severity; the likelihood that the individual had a prior history of a mental disorder is greater [14]. On the contrary, some studies found sleep disturbance precedes the depression in most cases [15]. For example, patients with insomnia are nearly 10 times more likely to develop depression than those without sleep complaints or whose insomnia had resolved [16,17]. Further, in a prospective study, the risk of developing major depression over twelve months was higher in individuals who had insomnia compared with those who did not. This risk was reduced if the insomnia resolved during the year [18]. This research would suggest that insomnia can be seen as a risk factor for the onset of a depressive episode [18,19]. The literature has currently not established the direction of causality; however, it is highly likely that the relationship is bi-directional. Therefore, any period where sleep is likely to be compromised is potentially a high risk time for the development of depression, setting up a vicious cycle of depressed mood and poor sleep.

Sleep is commonly disturbed during pregnancy and following childbirth [20]. Even individuals who have been ‘healthy sleepers’ prior to pregnancy will notice a change in their sleep patterns during pregnancy [21]. There are many physiological, psychological and lifestyle changes that occur during this period. Pain or discomfort while trying to sleep, and more frequent night-time toilet visits are common. Many women experience symptoms of insomnia, whilst some develop restless leg syndrome [22] and/or sleep apnoea [23]. Pregnancy requires adaptation to an ever-changing body and unfamiliar sleep patterns. As pregnancy progresses, complaints of sleep disruption become more prevalent [24] and sleep quality deteriorates significantly [25]. The most common sleep difficulties during pregnancy are frequent night wakening, difficulty falling asleep and waking too early. In fact, the vast majority of women (92%) report restless sleeping [26]. As a result, particularly if labour onset is at night or labour continues overnight, many women begin motherhood sleep deprived.

Sleep disturbances are common to all new mothers. The sleep patterns of new mothers are characterised by shorter sleep durations at night resulting in daytime fatigue [27,28]. This is particularly marked for first time mothers, who have lower sleep efficiency, spend more time in bed and have a greater wake after sleep onset during pregnancy than multiparous mothers [29]. These mothers also have fewer sleep episodes in the early postpartum period and generally poorer sleep quality than their multiparous counterparts [29,30]. The high rates of sleep disturbance amongst new mothers is not surprising given the many physical, lifestyle, psychological and emotional changes that occur following childbirth. During the first few days after childbirth, the breasts begin to produce milk, which is new and often uncomfortable. Genital, pelvic [31] and back pain [32] are also common after childbirth, which is known to further disturb sleep [33]. In addition to the physical changes, lifestyle changes and parenting responsibilities also contribute to the potential for sleep disruption. New infants sleep for short bursts, which gradually lengthen with time; but they generally do not sleep through the night for more than 5–6 hours before 6 months of age [34]. Hence, in addition to all the physical postpartum reasons for sleep to become interrupted, motherhood brings frequent, unavoidable night time wakening to attend to the baby’s needs. It has been suggested that sleep disruption may be one factor that contributes to a greater likelihood that women will become depressed in the postpartum period compared to other periods in their life [35]. However, there are surprisingly few studies that have investigated the relationship between sleep and PND.

Those studies that have assessed sleep and depression in the postpartum period, have confirmed strong associations [27], indicating that depressed mothers had poorer sleep quality, more sleep disturbance and more daytime sleepiness compared with non-depressed mothers. While there are few prospective studies, difficulty falling asleep or staying asleep predict PND [36]. Further, interventions that improve infant sleep patterns tend to see an improvement in maternal mental health although the cause and effect have not been confirmed [37,38]. If sleep disturbance can, in some cases, precede and even cause depressed mood amongst women during the postpartum period, then a sleep-focused intervention may have a role to play in preventing the development of mood disorders. However, there have been no investigations of the efficacy of interventions in the pre-natal period targeting both infant and postnatal maternal sleep.

Currently, there are a number of programs that exist to educate and prepare couples for childbirth and parenthood. The majority of programs educate parents-to-be about the practical and physical aspects of childbirth. While we were unable to find any programs with a major focus on sleep that occur prenatally, we did find a study examining a sleep intervention that occurs in the first few days following childbirth [39]. This study was conducted in Canada and randomised 30 new mothers to either an intervention or a control group. The intervention consisted of a private 45min consultation with a nurse which covered sleep hygiene, relaxation, strategies for increasing maternal sleep, acknowledgement of the challenges of parenting and sleep disruption, and details about infant sleep. They then received a booklet about sleep and had weekly phone calls for the first 5 weeks. The control group met the nurse for 10minutes, received a pamphlet about sleep and phone calls at 3 and 5 weeks postpartum. Outcomes for this study included questionnaires at baseline and 6 weeks postpartum as well as sleep diaries and actigraphy at 6 weeks postpartum. Results indicated an average of an extra 57 minutes of nighttime sleep in the intervention group than the control group. Fewer participants in the intervention group rated sleep as a problem than those in the control group and infants in this group had fewer night wakenings and longer nighttime sleeps than those in the control group. Despite the small sample size and limited power, the findings from this study are promising. As we were not able to find any large randomised controlled trials that occurred prenatally with a focus on sleep, the aim of this study is to bridge that gap.

This research will test the efficacy of a novel, brief sleep intervention for women during their final trimester of pregnancy using a cluster randomised design. The purpose of the intervention will be to educate women about what to expect in their sleep and their new baby’s sleep and how to adapt to these changes to reduce their sleep loss and manage their sleep better. We hypothesise that the intervention will result in improved scores on the primary outcomes, including sleep quality, sleep management and daytime sleepiness compared with the control condition. If the intervention is successful in improving sleep outcomes, we would expect that there would also be better outcomes observed in the mood of participants receiving the intervention.

### Aims and objectives

The aims of this study are:

1. To determine whether a sleep focused psychoeducational intervention delivered pre-natally can lead to better sleep outcomes for both mothers and infants during the post-natal period.

2. Given that insomnia and sleep disturbance can precipitate depression [40], if the sleep intervention does lead to better sleep outcomes in the postpartum period, we would like to determine whether this will reduce the likelihood of experiencing depressive symptoms.

### Hypotheses

1. Women who receive the sleep psychoeducational intervention will have better outcomes 6 weeks, 4 months and 10 months following delivery than those who do not receive the intervention on the following primary outcomes.

(a) Sleep quality – measured by the PSQI

(b) Sleep patterns – measured by shorter night wake periods indicated by the mother and infant sleep diary

(c) Self-efficacy – measured by the general self-efficacy

2. If changes in sleep, consistent with hypothesis 1(a,b) are observed, we would also expect that women who received the intervention will have fewer depressive symptoms at 6 weeks, 4 months and 10 months postpartum than the control group.

The PSQI is our primary outcome, which is a scale consisting of itemised scores which are likely to be affected differentially by pregnancy and motherhood. During pregnancy, the most common causes of disrupted sleep include the need to urinate, uncomfortable position, aching in a position, and being too hot or cold [26]. These common issues predominantly contribute to Item 5 and following the birth of the baby are generally normalised or reduced. With motherhood, women are woken several times during the night by the baby’s needs, and may develop different difficulties within Item 5. Sleep tends to deteriorate or remain unchanged for women as measured by the total PSQI. We expect this intervention will improve sleep outcomes relative to the control group.

### Outcomes and treatment effect

#### Primary outcomes

1. Mothers’ sleep quality indicated by:

• Pittsburgh Sleep Quality Index (PSQI)

• Insomnia Severity Index (ISI)

2. Mothers’ sleepiness indicated by:

• Multidimensional Assessment of Fatigue (MAF)

• Epworth Sleepiness Scale (ESS).

3. Sleep patterns: Sleep diaries and a Generic Sleep Questionnaire will help determine sleep patterns in the mother. This will indicate whether participants who receive the intervention have more consistent sleeping patterns or get more sleep than those in the control group or if the potentially different scores on the questionnaires are attributed to their ability to manage these changes better.

4. Actigraphy Measures

• Objective sleep-wake hours and patterns.

#### Secondary outcomes

1. Mothers’ depressive symptoms indicated by:

• Edinburgh Post Natal Depression Scale (EPDS)

• Depression, Anxiety and Stress Scale (DASS)

#### Process measures

There were a series of measures included in order to try and ascertain the mechanisms of treatment outcome. These are explained below:

1. Bonding between a mother and infant has high associations with poor sleep and low mood. Therefore a measure of bonding using the Being a Mother and Bonding Scale (BaMB) will be administered.

2. Coping skills mediate the relationship between stress and sleep [41] and are therefore an important measure to consider. We shall be assessing coping strategies using the Brief Coping Scale.

3. Infant temperament can have a profound effect on sleep opportunities for mothers. We shall control for this effect by measuring infant temperament with the Infant Characteristics Questionnaire.

4. The General Feeding Questionnaire includes night-time feeding patterns; which can often affect sleep.

## Methods

### Study design

This study will be a cluster randomised controlled trial. Participants will be recruited from consecutive attendees at prenatal classes at the Royal Prince Alfred Hospital (RPAH), Sydney Australia. In addition, the study is being advertised in obstetrician’s offices, and through the Study Facebook Page. Hence we will also accept self-referrals from these sources or from recommendations from friends. Participants from the same prenatal class will be randomised as a group in order to avoid contamination effects. The minimum in a cluster will be 3 and the maximum per cluster will be 10. Any participants from a group where less than 3 people consent or more than 10 people consent will join the next randomised cluster. Additional participants will join the clusters formed by the participants from RPAH based on the date they are recruited.

### Study population

Two hundred and fourteen first time mothers will be cluster randomised to a sleep psychoeducation intervention compared with a booklets only group (control). Women will be in their third trimester of pregnancy when recruited and will participate until 10 months after delivery. Fathers/partners will also be encouraged to attend the sleep intervention; however, women will not be excluded if their partners do not take part. Whether or not partners attend will be recorded. The inclusion criteria require participants speak sufficient English and do not have a history of major depression. Ethical approval was granted by the Ethics Review Committee of Sydney South West Area Health Service Sydney and is registered with the Australian New Zealand Clinical Trials Registry (ACTRN12611000859987).

### Data collection

Participants will have the option to complete their questionnaires either on paper or via the internet. Those who complete their questionnaires online will be allocated a unique link that will automatically save their questionnaires to the study’s secure server. They will be informed of their link at each time point. At the 6 weeks following delivery time point, additional data collection in the form of a phone call will occur. If participants have not completed their questionnaires within 2 weeks, they will be sent a reminder email. If another week passes and data has not been received, a second email will be sent. If participants still do not complete their questionnaires, they will receive a call from the research coordinator who will collect their data over the phone. There will be an option for the participants to fill in their sleep diaries through a mobile app which is user friendly and similarly confidential.

## Materials

The Baseline questionnaires include:

1. Pittsburgh Sleep Quality Index (PSQI)

2. Insomnia Severity Index (ISI)

3. Multidimensional Assessment of Fatigue (MAF) scale

4. Epworth Sleepiness Scale (ESS)

5. Edinburgh Post Natal Depression Scale (EPDS)

6. Depression, Anxiety and Stress Scale (DASS)

7. Brief Coping Scale (COPE)

8. The General Self-Efficacy Scale (SES)

Post-delivery questionnaires include the baseline questionnaires as well as the questionnaires listed below:

9. Infant characteristics questionnaire (ICQ)

10. General feeding questionnaire

11. General sleeping questionnaire

12. Being a Mother & Bonding Scale – BaMB

13. Maternal sleep diary

14. Infant sleep diary

A description of all the questionnaires these can be found in Appendix: Questionnaires.

The Maternal and Infant sleep diaries are designed to record sleep hours for three consecutive days. There will be the option to complete these via a specially designed phone application instead of online for those who find it more convenient.

In the interest of efficiency and in order to minimise the number of questionnaires completed by the participants, the Brief COPE questionnaire, The Infant Characteristics Questionnaire and the General Feeding Questionnaire will not be administered at the 4 month time points. Furthermore, the 10 month time point will utilise the questionnaires related to the primary and secondary outcomes only.

An actiwatch will also be used for selected participants. An actiwatch is a wrist-like device which monitors movement above a given threshold via an accelerometer and provides data on sleep-wake patterns over a period of time [42].

### Procedure

#### Recruitment from Royal Prince Alfred Hospital (RPAH) prenatal classes

Patients attending prenatal classes at the RPAH will be approached by the study coordinator with a brief explanation of the study and the participant information sheet. Participants will be given the opportunity to provide their contact details. The study coordinator will then make contact with these individuals to explain the study more thoroughly and screen them for eligibility.

#### Recruitment from Facebook.com

In order to maximise recruitment, a Facebook page was published, facilitating participants to ‘share’ or recommend the study with other potential participants as well as allowing interested individuals to access the information and volunteer themselves as participants. The link to the active Facebook page as well as a screen shot of the page and the pictures presented on the page can be found in Additional file 1: Figure S2: *Facebook Page ‘Sleep for New Mums’.*

#### Time 1

Participants will be called by the study coordinator. The study will be explained and medical history, demographics and other relevant information will be collected. If the individuals are eligible, they will be asked to provide informed consent and complete the set of baseline questionnaires.

#### Randomisation

Envelopes have been prepared and sealed by an independent researcher. Each enveloped is labeled ‘Cluster1’ (C1), ‘Cluster2’ (C2) etc.… Once a minimum of 3 and a maximum of 10 participants have been screened and completed the *Time 1* components, they will be allocated into a cluster and an envelope will then be opened to reveal whether the participants in that cluster will receive intervention or control condition.

#### Time 2

Participants will receive a phone call from the study coordinator 3 weeks following the birth of the baby. For the intervention group, this is when potentially bad habits may start to form and as such we aim to remind the participants of the intervention and its content. For the control group, the phone call will be simply to provide support, which might otherwise vary between the groups.

#### Time 3

Six weeks after delivery, participants will be contacted by phone. The phone call will be scripted involving questions about sleep hours and patterns as well as courtesy questions about how they are managing. Participants in the intervention group will receive an additional segment of the script reminding them to look through the notes provided during the program and offering advice and support if necessary. We shall explore what has or has not been useful for them and direct them to the relevant section of their notes to review. We will also allow them to ask any questions they may have about sleep. At this stage, all participants will complete the online questionnaires once again. These questionnaires will include the baseline and post-delivery questionnaires.

#### Time 4

Approximately 4 months after delivery, participants will fill out the baseline and post-delivery questionnaires again.

#### Time 5

**Figure 1:**
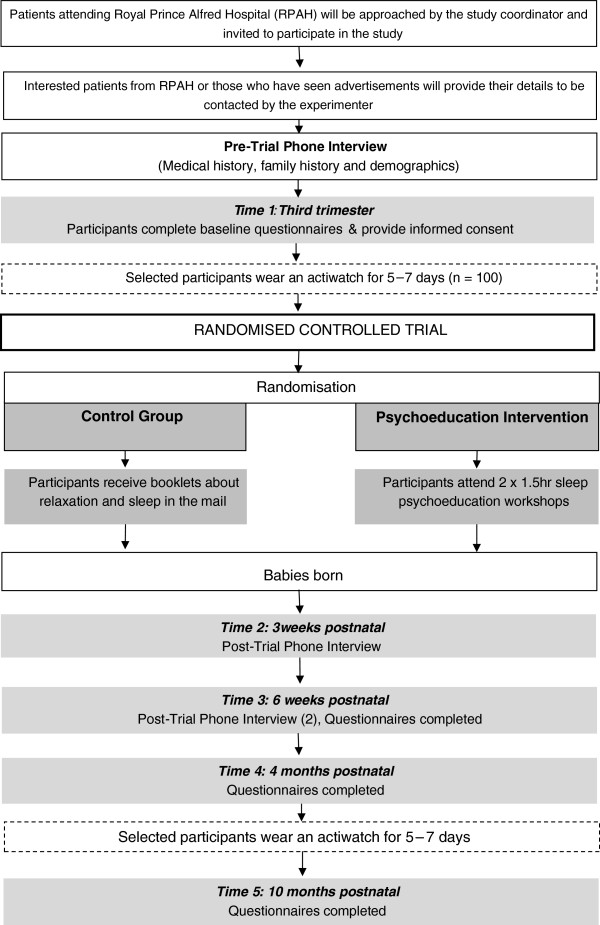
Procedure flowchart.

10 months after delivery; participants will fill out 2 questionnaires about sleep (PSQI & ISI), 2 about depression (DASS & EPDS) and the sleep and feeding questionnaires. This will conclude their participation in the study. A flowchart of the procedure can be seen in Figure 1.

The time points for data collection have been designed to follow common milestones in sleep pattern developments and changes. *Time 3 (6 weeks postpartum)* has been scheduled as few infants develop a consistent sleep wake pattern before this time so we will be able to explore how the mothers have managed this inconsistency as well as being able to give them guidance about what to look for over the coming weeks as the patterns do start to emerge [43]. *Time 4 (4 months postpartum)* has been scheduled as infants begin to develop the self-soothing abilities around 4 months of age [44] and it is at this time that the most rapid consolidation of sleep regulation occurs [45]. Therefore, completing the questionnaires at this time point will indicate changes when infants have a more predictable sleep wake pattern. *Time 5 (10 months postpartum)* has been scheduled for two reasons. Firstly, most infants no longer have a nutritional need for a night time feed and therefore in most instances babies should be able to sleep through the night at this stage [45]. Secondly, assessing mood at this time will ensure that the majority of participants with postnatal depression are found as most postnatal depression onset occurs by the 10^th^ month postpartum [45,46].

Two participants per cluster will be chosen to wear an actiwatch in order to collect actigraphy measures for 5 – 7 days at *Time 1* and 5 – 7 days at *Time 4*. The actigraphy measure will be an objective indicator of sleep-wake patterns and a validation of their self-reported sleep patterns. The actiwatches will be posted to selected participants with a reply-paid envelope. For practical reasons, the participants in each group whose babies have the latest due date will be asked to participate in the actigraphy study.

### Intervention

The intervention will consist of two 1.5hrs psychoeducational sessions held in the evenings at institutes connected to The University of Sydney. The first session, will focus on the sleep of the mother and the topics below will be outlined:

1. Understanding Sleep

2. Sleep changes during pregnancy

3. Adjusting to sleep disruption with parenthood

4. Expectations with becoming a parent

5. Sleep and Mood

6. Sleep during labour

7. Sleep for the first few days & weeks

8. Sleep and anxiety

9. The role of the partner

10. Understanding fatigue

11. Managing fatigue… as best you can

The second session will focus on the sleep of the baby. The topics below will be covered:

1. Foetal Sleep

2. Infant Sleep (quiet and active)

3. Baby monitors

4. How sleep needs change with age

5. How to know when your baby needs sleep

6. Basic rest & activity cycle (BRAC)

7. Helping your baby get to sleep & Soothing strategies

8. N.A.P.S. (Note time, Add 90, Play, Soothe)

9. Infant sleep 3 – 12 months

The sleep intervention contains videos of three new mothers with babies aged 6 weeks, 5 months and 6 months. These ‘real life’ mothers share their experiences of motherhood from their sleep & mood to how they learned to feed/settle their babies. A key message was the variation in their individual experiences of motherhood.

The program seeks to normalise sleep changes that occur during pregnancy and as a new mother. Further, it will encourage women to develop strategies to improve sleep when necessary. Specifically, information on the mechanisms of sleep and the importance of circadian rhythm and how this develops in newborns will be provided. Specific attention will be given to the difference between active and quiet sleep in newborns to ensure that new mothers do not mistake the transition between sleep stages and disrupt sleeping infants unnecessarily. Furthermore, we aim to clarify the many misconceptions associated with sleep and in particular, napping. Following the workshop, participants will have information of how to manage naps in order to improve daytime fatigue without disrupting their subsequent night of sleep.

The program aims to reassure women, that while they may get very little sleep in the first few weeks, infants will make changes in their sleep patterns as they develop. Hence, the sleep disturbance associated with motherhood will improve with time. During these early times, where sleep is necessarily compromised, the program will encourage women to sleep in preference to other activities, such as completing housework or socialising [47], when necessary.

Although the individuality of different infants will be emphasised, common infant signals for sleepiness and settling strategies will be discussed. As part of the educational component, the program describes common sleep patterns and milestones at different ages of the infant so that new mothers are prepared for the typical patterns of sleeping that characterise different stages of infancy. We also include interviews with new mothers to emphasize the key educational points raised.

The relationship between mood and sleep disturbance will be highlighted. The program will encourage women to enlist help from their partner, family and friends during this time.

The control group will receive booklets about babies’ sleep, managing sleep long term and relaxation strategies. These booklets will be given to the intervention groups as well.

### Statistical considerations

Power is difficult to calculate given that only one study has attempted to intervene in sleep in the pre or postnatal period. That intervention was delivered early in the postpartum period. The authors found a large effect size on both sleep duration (ES = 1.15), and depressive symptoms using the Edinburgh Postnatal Depression Scale (ES= 0.78) [39]. It is unclear whether a preventative program would achieve such large effect sizes. However, studies in individuals with insomnia attending a sleep intervention have consistently found effect sizes in the 0.4 – 0.8 range [48,49]. Hence, we chose the smallest of these effect sizes in order to be conservative. This was particularly important as the effect sizes we have taken into consideration were shown in interventions that treated people for insomnia, which one would expect to have a greater effect than a preventative program wherein the sample begins at a baseline level. The effect sizes (Cohen’s d) from past sleep programs using the sleep questionnaires of the current study (the PSQI and ISI) has ranged from 0.35 – 0.88 [50]. On the basis of these in order to achieve 80% power, based on the most conservative effect size, we would need only 41 participants per group to achieve significance at 0.05 level. However, the cluster design increases the sample size requirement according to the following formula: 1 + (*m* – 1)*ICC, where ICC is the intra-cluster correlation, (the correlation between patients’ scores within each group) and *m* is the average number of patients per group. We could not find any published studies, using the sleep measures and reporting ICCs. Therefore, we based our assumptions on ICCs reported for mood variables. Again, we could not find any for EPDS, however, Adams et al. (2004) [51] published 1039 ICCs from 31 studies and found the median value to be 0.01 for the HADS (interquartile range [IQR] 0 to 0.032, range 0 to 0.840). We aim for an average group size of 6, and to be conservative took the mean for the ICC of 0.42, requiring 2.6 times the number of participants. Therefore to ensure sufficient power to find differences on quality of life at an effect size of at least 0.4, we need 107 participants per group (n=214).

We anticipate that the support from the Royal Prince Alfred Hospital as well as the Facebook page that has been set up to accept anyone who expresses interest, will enable us to recruit a sample of 214 participants over 1.5years. We have aimed to be strategically conservative in calculating sample size.

### Data analysis plan

There are 4 measurement time points, of which 3 are post-intervention (6 weeks, 4 month, and 10 months follow up). A linear mixed model analysis will be implemented for each factor (sleep quality and depression) at each time point. This method takes account of both fixed and random effects and is robust to account for missing data. Fixed effects will include time; (baseline, 6 week, 4 months, 10 months) and group (intervention and control). Random effects will include subjects to account for between-subject variation. Uncorrected pairwise comparisons based on estimated marginal means will evaluate the primary hypothesis of interest – that the sleep quality of the participants in the intervention group will decline less in the postpartum period than those in the control group. Comparisons of baseline measures with post treatment will be used at follow up compared with controls.

## Discussion

Sleep is one of only a few modifiable risk factors for postpartum depression. The aim of the current study is to provide women with information about maternal and infant sleep and how to manage sleep optimally. If mothers who receive the psychoeducational intervention can achieve better sleep than those who do not, this program has the potential (by decreasing a risk factor for postpartum depression) of reducing the risk of developing depressive symptoms during the postpartum period.

### Strengths

This study will test the efficacy of a novel psychoeducational intervention that targets sleep in first time mothers. The intervention has been developed by a midwife and psychologist (Delwyn Bartlett) with experience working with patients with insomnia and a clinical psychologist experienced in the treatment of depression (Louise Sharpe). The program is supported by a PowerPoint presentation to ensure accuracy across groups and will be administered by a psychologist (Liora Kempler).

The trial will be conducted in accordance with the CONSORT guidelines and their extension to cluster-randomised controlled trials [52]. Women from a large, teaching hospital as well as interested women from Sydney, Australia will be consecutively recruited to the study and randomised (in clusters) to receive either the intervention in addition to their routine prenatal care, or their routine care only. Hence, at the completion of the study, the intervention will be easily translated into routine clinical care of all new mothers. The use of a range of outcome measures at baseline and six weeks, four months and ten months postpartum will ensure that the full range of benefits of the program can be documented. Further, it will allow the mechanisms of treatment to be established.

### Challenges

The most significant challenge that we expect to have is recruitment and the rate of attrition. We have attempted to combine the groups as closely as possible with routine prenatal care and to offer women a range of options in how to complete the questionnaires at each assessment point. In addition, to thank women for their time in participating in the study, we will offer participants the chance to win a hamper of baby goods worth $200 upon completion of their 10 month follow up questionnaires.

### Relevance

If the current study produces positive results, it would be easily adapted into wider practice. Most women routinely access post-natal classes and this intervention could easily be added to those classes as part of routine care. Furthermore, if improving sleep can reduce depressive symptoms, the program could be offered as a preventative measure for postnatal depression and could be offered to women at risk of developing postnatal depression such as those with a history of depression.

## Conclusion

In summary, this study has the potential to produce evidence demonstrating an educational intervention can have a preventative effect on sleep disturbance and mood disorders in the postpartum period.

## Trial status

Recruitment of participants is in progress.

## Appendix

Questionnaires

1. *The Pittsburgh Sleep Quality Index (PSQI)* has 11 questions and is widely used in sleep research to assess sleep quality, insomnia symptoms, medication use and other sleep disorders.

2. *The Insomnia Severity Index (ISI)* measures experience with sleep onset and sleep maintenance.

3. *The Multidimensional Assessment of Fatigue (MAF)* scale measures levels of fatigue.

4. *The Epworth Sleepiness Scale (ESS)* asks participants to rate their chance of dozing in certain circumstances.

5. *The Depression, Anxiety and Stress Scale (DASS)* is a four point scale assessing subjective depression, anxiety and stress.

6. *The Edinburgh Post Natal Depression Scale (EPDS)* is the most widely used questionnaire to assess post natal depression.

7. *The Being a Mother & Bonding Scale (BaMB)* assesses the experience of motherhood and the mother-infant relationship.

8. *The Brief Coping Scale (COPE)* assesses coping strategies in reference to operations but can be applied to a number of new experiences.

9. *The Self Efficacy Scale (SES)* rates ones belief in their abilities to do new things and manage new experiences.

10. *The Infant Characteristics Questionnaire (ICQ)* assesses subjective infant temperament and general behaviour.

11. *The General Feeding and Sleeping Questionnaires* have been designed specifically for this study to examine the number of hours spent feeding the baby and sleeping.

12. *The Maternal and Infant Sleep Diaries* measure subjective sleep patterns of the mother and infant over 3 days.

## Abbreviations

BaMB: Being a Mother and Bonding Scale; CONSORT: Consolidated Standards of Reporting Trials; DASS: Depression Anxiety and Stress Scale; EPDS: Edinburgh Post Natal Depression Scale; ESS: Epworth Sleepiness Scale; SES: General Self Efficacy Scale; ICQ: Infant characteristics questionnaire; ISI: Insomnia Severity Index; MAF: Multidimensional Assessment of Fatigue; PSQI: Pittsburgh Sleep Quality Index; PND: Postnatal Depression; RPAH: Royal Prince Alfred Hospital.

## Competing interests

All authors declare that they have no competing interests.

## Author’s contributions

LK drafted the manuscript and all authors were involved in the design and revision of the manuscript. DB is the grant holder. All the authors read and approved the final manuscript.

## Pre-publication history

The pre-publication history for this paper can be accessed here:

http://www.biomedcentral.com/1471-2393/12/155/prepub

## Supplementary Material

Additional file 1 **Figure S1. **Facebook Page ‘Sleep for New Mums’ Screen Shot. **Figure 2:** Facebook Page ‘Sleep for New Mums’. Link: http://www.facebook.com/pages/Sleep-for-New-Mums/438015452906586?fref=ts. Click here for file
