# Supplementary material for: Sleep education during pregnancy for new mothers
Source: BMC Pregnancy Childbirth. 2012 Dec 17;12:155. doi: 10.1186/1471-2393-12-155 (PMC3546917; doi:10.1186/1471-2393-12-155)

Appendix 2, Figure 1: Facebook Page 'Sleep for New Mums' Screen Shot

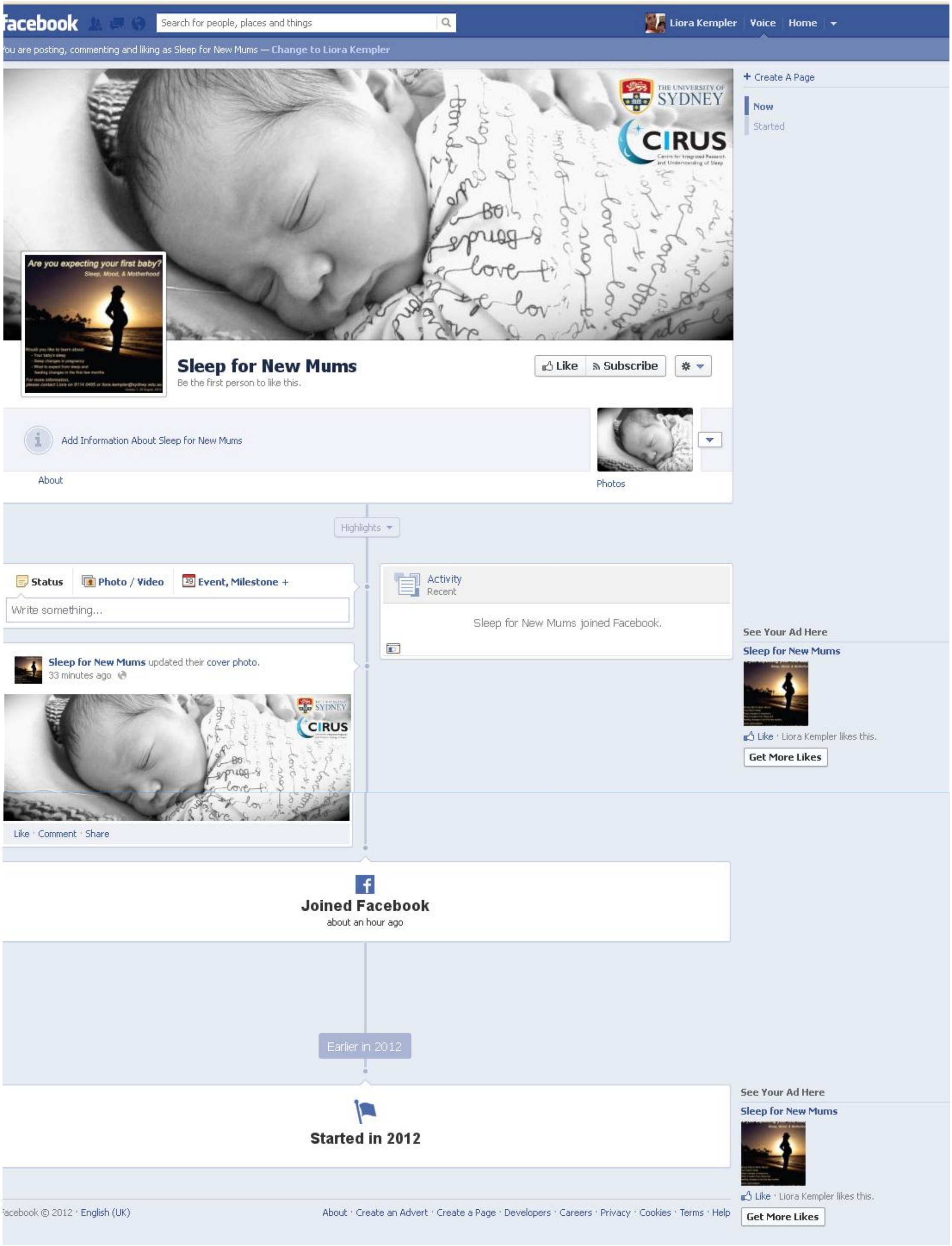

Supplement: Additional file 1 — Figure S1. Facebook Page ‘Sleep for New Mums’ Screen Shot. Figure 2: Facebook Page ‘Sleep for New Mums’. Link: http://www.facebook.com/pages/Sleep-for-New-Mums/438015452906586?fref=ts. [file 1471-2393-12-155-S1.pdf]
